# Exploring the Efficacy of Peptides and Mimics against Influenza A Virus, Adenovirus, and Murine Norovirus

**DOI:** 10.3390/ijms25137030

**Published:** 2024-06-27

**Authors:** Umme Laila Urmi, Ajay Kumar Vijay, Mark D. P. Willcox, Samuel Attard, George Enninful, Naresh Kumar, Salequl Islam, Rajesh Kuppusamy

**Affiliations:** 1 School of Optometry and Vision Science, University of New South Wales, Sydney, NSW 2052, Australia; m.urmi@unsw.edu.au (U.L.U.); v.ajaykumar@unsw.edu.au (A.K.V.); g.enninful@unsw.edu.au (G.E.); salequl@juniv.edu (S.I.); rajesh.kuppusamy@sydney.edu.au (R.K.); 2 School of Chemistry, University of New South Wales, Sydney, NSW 2052, Australia; s.attard@unsw.edu.au (S.A.); n.kumar@unsw.edu.au (N.K.); 3 Department of Microbiology, Jahangirnagar University, Savar 1342, Bangladesh; 4 The Drug Discovery Initiative, University of Sydney, Sydney, NSW 2006, Australia

**Keywords:** peptide, peptidomimetics, influenza A virus, adenovirus, norovirus

## Abstract

The ongoing battle against viral pandemics continues, with the possibility of future outbreaks. The search for effective antiviral compounds that can combat a diverse range of viruses continues to be a focal point of research. This study investigated the efficacy of two natural antimicrobial peptides (AMPs) (lactoferricin and LL-37), two synthetic AMPs (melimine and Mel4), and nine AMP mimics (758, 1091, 1096, 1083, 610, NAPL, 3-BIPL, 4-BIPL, and Sau-22) against influenza A virus strains H1N1 and H3N2, human adenovirus 5 (HAdV-5), and murine norovirus 1 (MNV-1). These compounds were tested using virus pre-treatment, cell pre-treatment, or post-cell entry treatment assays, electron microscopy, and circular dichroism (CD), alongside evaluations of cytotoxicity against the host cells. After virus pre-treatment, the AMP mimics 610 and Sau-22 had relatively low IC_50_ values for influenza strains H1N1 (2.35 and 6.93 µM, respectively) and H3N2 (3.7 and 5.34 µM, respectively). Conversely, natural and synthetic AMPs were not active against these strains. For the non-enveloped viruses, the AMP Mel4 and mimic 1083 had moderate activity against HAdV-5 (Mel4 IC_50_ = 47.4 µM; 1083 IC_50_ = 47.2 µM), whereas all AMPs, but none of the mimics, were active against norovirus (LL-37 IC_50_ = 4.2 µM; lactoferricin IC_50_ = 23.18 µM; melimine IC_50_ = 4.8 µM; Mel4 IC_50_ = 8.6 µM). Transmission electron microscopy demonstrated that the mimics targeted the outer envelope of influenza viruses, while the AMPs targeted the capsid of non-enveloped viruses. CD showed that Mel4 adopted an α-helical structure in a membrane mimetic environment, but mimic 758 remained unstructured. The diverse activity against different virus groups is probably influenced by charge, hydrophobicity, size, and, in the case of natural and synthetic AMPs, their secondary structure. These findings underscore the potential of peptides and mimics as promising candidates for antiviral therapeutics against both enveloped and non-enveloped viruses.

## 1. Introduction

**Figure 1:**
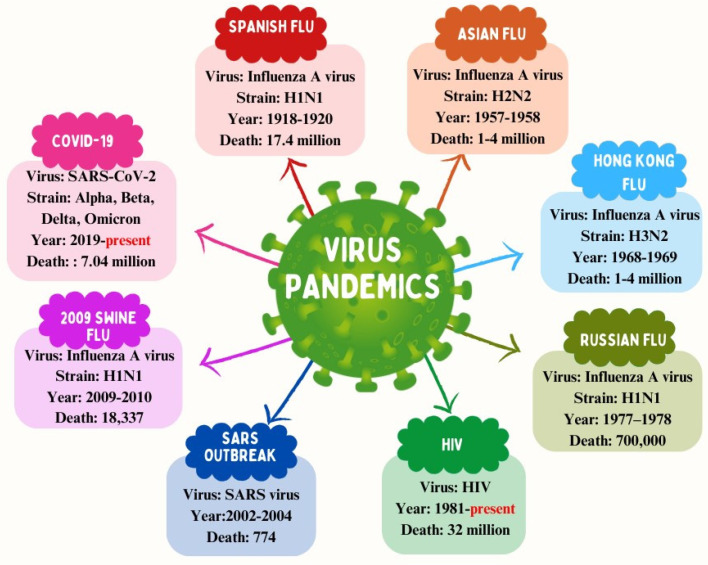
Major twentieth- and twenty-first-century viral pandemics.

Throughout the course of human history, viral infections have been a persistent challenge for global health. Various epidemics highlight the severity of these challenges, such as the Spanish flu (H1N1) from 1918 to 1920, with a death toll of 17.4 million [1], Asian flu (H2N2) in 1957–1958, causing 1 to 4 million deaths [2], Hong Kong flu (H3N2) in 1968–1969, resulting in 1 to 4 million deaths [3], Russian flu (H1N1) in 1977–1978, causing 700,000 deaths [4], swine flu (H1N1) in 2009, with 18,337 deaths [5], the ongoing HIV pandemic, which, since 1981, has claimed at least 32 million lives [6], and the ongoing COVID-19 pandemic (SARS-CoV-2), which, since 2019, has claimed at least 7 million lives (Figure 1) [7].

Influenza, a member of the Orthomyxoviridae family, is an enveloped single-stranded negative-sense RNA virus [8] capable of producing viral particles with diverse shapes, including spherical, bacillary, or filamentous types [9]. It is categorized into four genera: influenza A (IAV), influenza B, influenza C, and influenza D [8]. All documented occasional influenza pandemics and seasonal epidemics result from infections caused by IAV. The emergence of pandemic IAV variants typically involves processes known as “antigenic shift” (slightly modified genes) and “antigenic drift” (entirely new genes) [10]. Vaccines have played a key role in preventing IAV infections. However, the creation of vaccines to counter new strains is a time-consuming process. While there are some drugs designed to treat influenza, such as oseltamivir or zanamivir, over time, the viruses can develop resistance to these drugs [11]. Consequently, there is a need for the development of new anti-influenza drugs.

Human adenoviruses (HAdVs) are non-enveloped with double-stranded DNA [12]. They primarily spread through nasal and fecal–oral routes [13]. As they lack an envelope, they are remarkably durable in harsh conditions and are poorly susceptible to chemical disinfectants and sanitizers [14,15]. Adenovirus infections can manifest in diverse forms, including respiratory [16,17], ocular [18,19], and enteric infections [20,21], with respiratory infections sometimes being fatal. Cases of adenoviral epidemic keratoconjunctivitis occur through contaminated ophthalmic instruments and eye solutions, hand-to-eye contact by infected personnel, or from swimming pools [22]. Adenovirus outbreaks have been documented in various settings, such as hospitals, neonatal intensive care units, and university campuses, around the world [23,24,25,26,27]. Despite the seriousness of this virus, there are currently no approved treatments specifically designed for adenovirus. Instead, antiviral medications developed for other DNA/RNA viruses are used to manage infections [28]. Although, in recent years, several new compounds and formulations have been shown to combat HAdV in laboratory and animal experiments [28,29,30], they have yet to be brought to the market. Consequently, there is a demand for improved anti-adenoviral therapies.

Human norovirus (HuNoV), a Norwalk-like virus, is a positive-sense, single-stranded, non-enveloped RNA virus with a diameter ranging from approximately 27 to 35 nm, belonging to the Caliciviridae family [31]. Transmitted through the fecal–oral pathway [32], HuNoV is a prevalent enteric pathogen responsible for viral gastroenteritis and is the primary cause of nonbacterial gastroenteritis [32]. Numerous gastrointestinal outbreaks caused by this virus have been reported worldwide [33,34,35,36]. Since it leads to diarrhea, the primary treatment involves supportive fluid therapy [37], as there are no FDA-approved drugs specifically targeting norovirus [37]. Compounds that target its protease are potential therapeutics [38,39], but these have not been brought to the market. Consequently, the development of an effective and safe norovirus therapeutic or prophylactic antiviral is required.

Antimicrobial peptides (AMPs) can be antiviral, having efficacy against various enveloped and non-enveloped viruses [40]. These peptides have a broad spectrum of activity and exhibit diverse modes of action. For instance, LL-37 is effective against HIV, coronaviruses, zika virus, dengue virus, vaccinia virus, aichi virus, and human enterovirus through distinct mechanisms [40]. Similarly, other peptides such as HNP-1, HNP-2, Protegrin 1, and mucroporin-M1 can have wide-ranging antiviral activity against various viruses [40]. Despite their significant potential as novel antiviral agents, AMPs face several challenges that limit their practical application. One major limitation is their rapid degradation in vivo, as they are highly susceptible to proteolytic cleavage by enzymes in the body [41]. Additionally, AMPs often have limited bioavailability because they tend to bind to plasma proteins, reducing their effective concentration in the bloodstream [42]. Furthermore, the production of AMPs is often costly, which can be a significant barrier to their widespread use and development as therapeutic agents [41]. Addressing these challenges is crucial for the successful translation of AMPs from experimental compounds to practical antiviral therapies.

Peptidomimetics of AMPs can be prepared by altering an existing peptide or designing molecules that have structural properties similar to AMPs [43]. These modifications often involve altering the chemical structure to enhance stability and efficacy. One significant advantage of peptidomimetics is their resistance to proteolytic cleavage, which addresses a major limitation of traditional AMPs that are prone to rapid degradation by enzymes in the body [44]. Moreover, peptidomimetics are generally more cost-effective to produce compared to natural peptides, making them a more feasible option for large-scale production and therapeutic use. Small molecule peptidomimetics based upon glyoxamide, tryptamine, or anthranilamide have been shown to have suitable antibacterial activities [45,46,47,48,49,50,51,52]. Specifically, anthranilamide-based peptidomimetics have shown promising antiviral properties, with documented efficacy against pathogens like Herpes simplex virus and certain coronaviruses [53]. This ability to retain or even enhance the biological activity of AMPs while also improving stability and reducing production costs underscores the potential of peptidomimetics as a versatile and powerful tool in the development of new antimicrobial and antiviral therapies.

Therefore, due to the increasing resistance against current anti-influenza agents and the lack of specific treatments for adenoviruses and noroviruses, our study evaluated a diverse array of compounds, including natural and synthetic peptides, as well as peptide mimics (such as anthranilamide-based mimics) for their antiviral properties. Consequently, our research has the potential to pave the way for the development of new antiviral therapies that are more robust against drug resistance and viral mutations. Such advancements would provide a crucial tool in the fight against current and future viral pandemics, offering broader and more reliable protection.

## 2. Results

This study examined thirteen compounds, including two natural peptides (LL-37 and lactoferricin), two synthetic peptides (melimine and Mel4), and nine peptide mimics (758, 1091, 1096, 1083, 610, NAPL, 3-BIPL, 4-BIPL, and Sau-22). The overall experiment aimed to explore the activity and mechanism of action of these compounds against H1N1, H3N2, HAdV-5, and murine norovirus type 1 (MNV-1). The nine peptide mimics have not previously been examined for activity against influenza virus, adenovirus, or norovirus. While a previous study had shown that compounds with similar backbone anthranilamide structures (called compounds 11 and 14 in the previous study) [53] were active against the enveloped viruses Herpes simplex I and mouse hepatitis virus (a coronavirus), this study for the first time examined whether the anthranilamide structures were active against non-enveloped viruses (adenovirus and norovirus), as well as influenza viruses.

### 2.1. Direct Inactivation

#### 2.1.1. Antiviral Effect on H1N1

**Figure 2:**
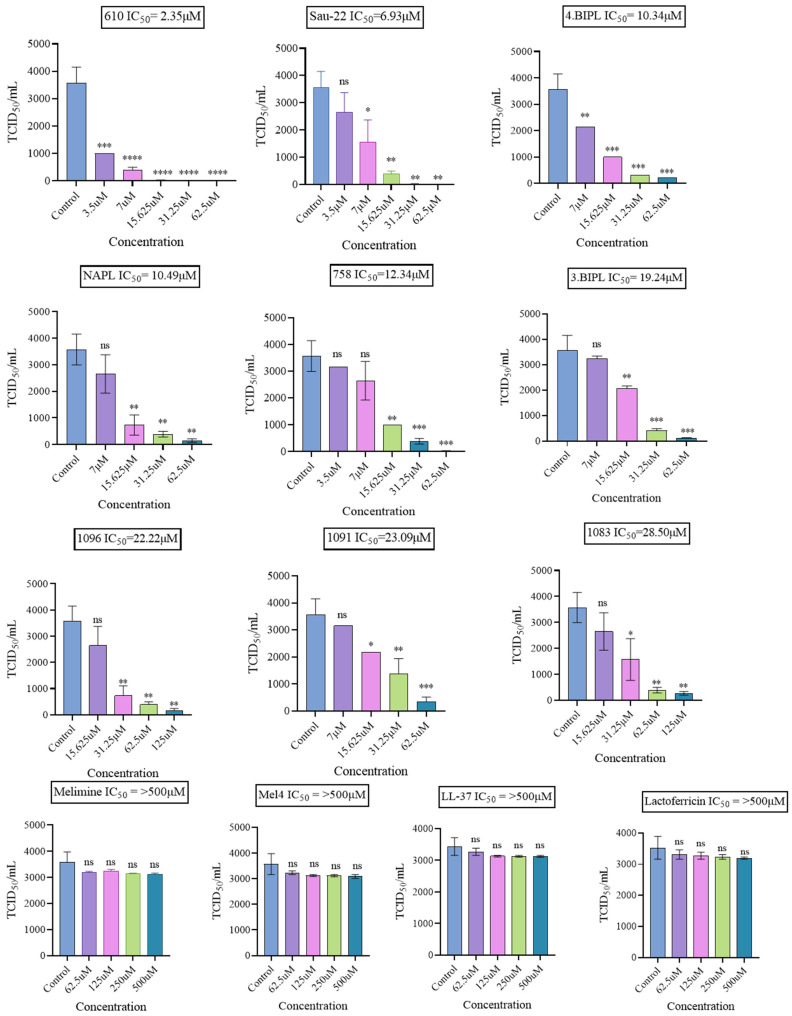
The ability of peptide mimics to reduce the infectivity of Influenza virus H1N1. Graphs show the TCID_50_ values of each peptide mimic at different concentrations, and non-linear regression analysis was used to determine the IC_50_ for each peptide. * *p* < 0.05. ** *p* < 0.01. *** *p* < 0.001. **** *p* < 0.0001. ns, not significant.

Initially, a direct inactivation method was employed to assess all compounds, where 3 × 10^3^ TCID_50_/mL of H1N1 was incubated with varying concentrations of the test compounds for 3 h. In this test, none of the natural or synthetic peptides demonstrated any activity, even at concentrations as high as 500 μM (Table 1). Conversely, all tested mimics exhibited antiviral activity, with the lowest IC_50_ values occurring with 610 (IC_50_ = 2.35 μM) and Sau-22 (IC_50_ = 6.93 μM; Figure 2 and Table 1). To assess the cytotoxicity of active antiviral compounds on the host MDCK cells, an MTT assay was conducted, followed by the calculation of CC_50_ (Table 1) to determine the selectivity indices (SI; Table 1). SI was calculated by determining the ratio of CC_50_ to IC_50_, with higher numbers indicating relatively safe compounds. Based on the SI values, compounds with SI ≥ 10 were 610 (SI = 14.1), 758 (SI = 11.9), Sau-22 (SI = 11.4), and NAPL (SI = 10.6; Table 1).

#### 2.1.2. Antiviral Effect on H3N2

**Figure 3:**
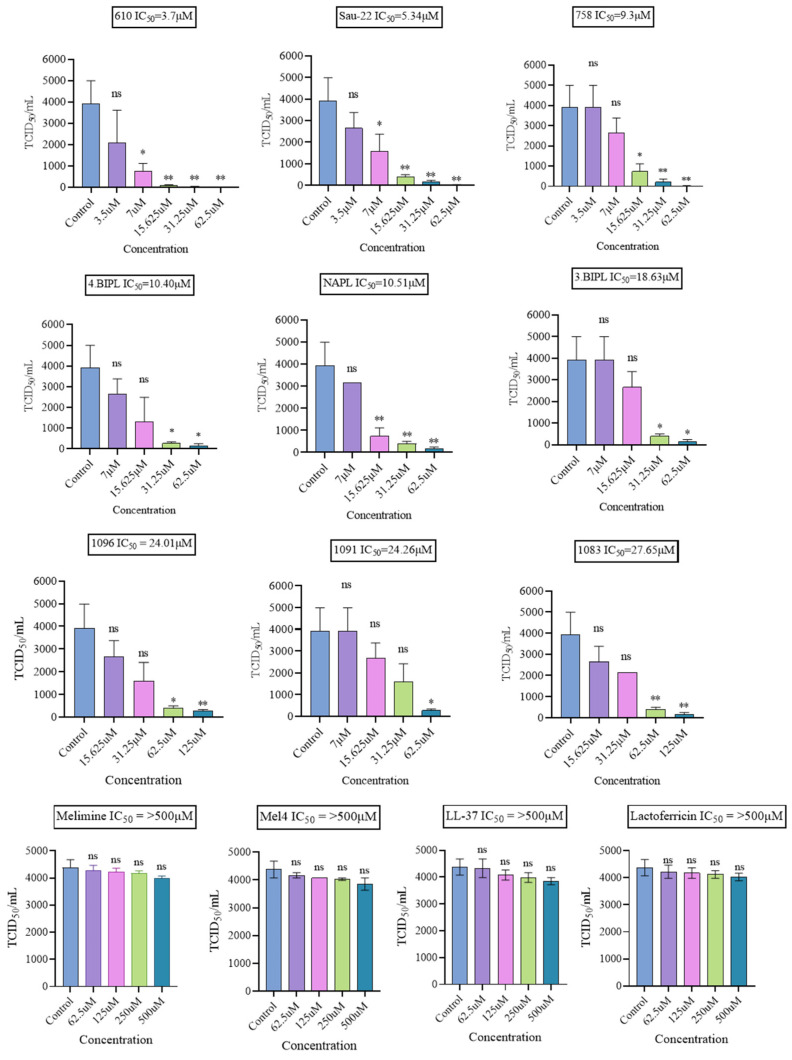
The ability of peptide mimics to reduce the infectivity of influenza A virus H3N2. Graphs show the TCID_50_ values of each peptide mimic at different concentrations, and non-linear regression analysis was used to determine the IC_50_ for each peptide. * *p* < 0.05. ** *p* < 0.01. ns, not significant.

In the subsequent experiment, these compounds were evaluated against another influenza strain, H3N2, using a similar assay as above. Similar outcomes were observed for all the compounds against this strain as well. Natural and synthetic peptides exhibited no antiviral activity against H3N2 (Table 1), while the peptide mimics demonstrated potent antiviral activity. Figure 3 illustrates the overall impact of each compound and concentration on H3N2. IC_50_ values were calculated, and 610 and Sau-22 continued to exhibit the most robust antiviral activity with IC_50_ values of 3.7 μM and 5.34 μM, similar to those observed for H1N1 (Table 1). SI values were determined, and a summary of antiviral activity, cytotoxicity, and selectivity indices is presented in Table 1. The same compounds, as observed with H1N1, emerged as potent anti-H3N2 agents based on SI values: 758 (SI = 15.8), Sau-22 (SI = 14.8), NAPL (SI = 10.6), and 610 (SI = 9), as well as 1083 (SI = 9.05).

#### 2.1.3. Antiviral Effect on the Non-Enveloped Viruses HAdV-5 and MNV-1

**Figure 4:**
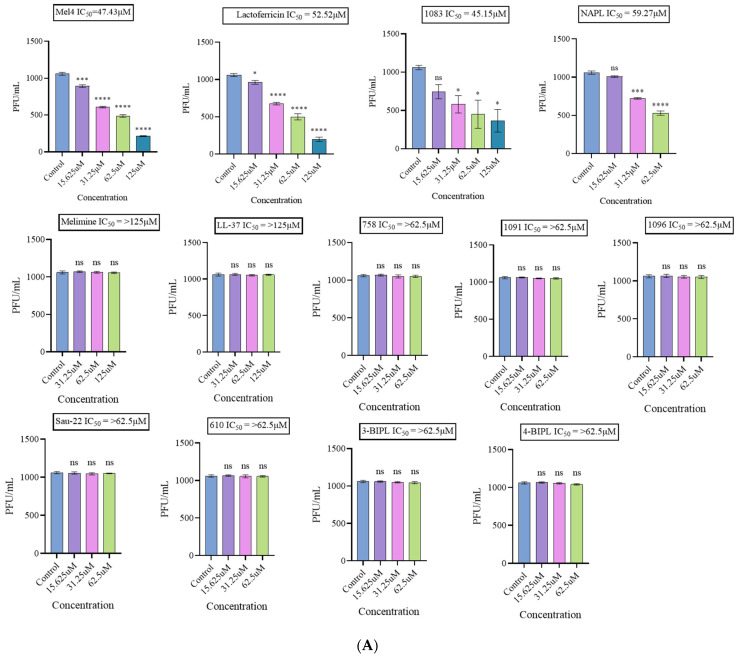

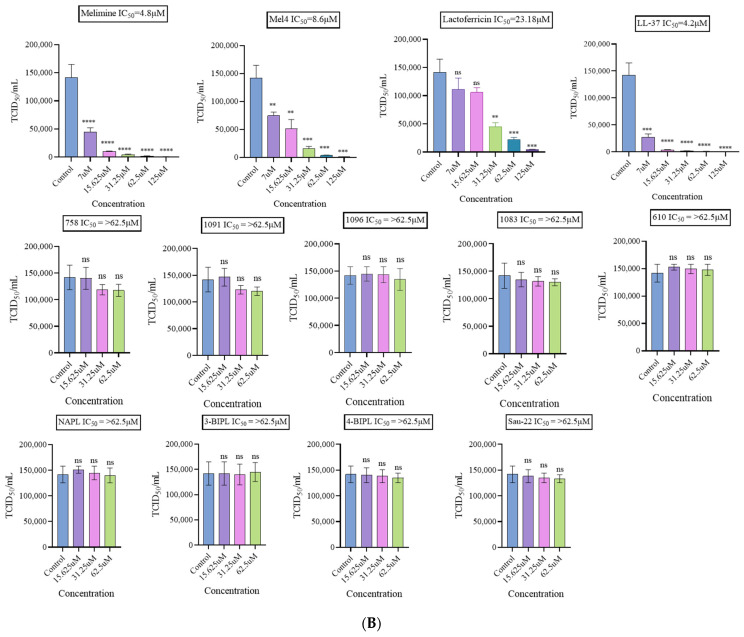
The ability of peptide mimics to reduce the infectivity of human adenovirus type 5 (**A**) and murine norovirus 1 (**B**). Graphs show the TCID_50_ values of each peptide mimic at different concentrations, and non-linear regression analysis was used to determine the IC_50_ for each peptide. * *p* < 0.05. ** *p* < 0.01. *** *p* < 0.001. **** *p* < 0.0001. ns, not significant.

After incubating the peptides and mimics for 3 h, their effectiveness against HAdV-5 was found to be limited. Among the peptides, the IC_50_ for lactoferricin was 52.52 µM, and for Mel4 was 47.43 µM. Among the mimics, only 1083 and NAPL showed limited activity, with IC_50_ values of 45.15 µM and 59.27 µM, respectively (Figure 4A). The cytotoxicity profile and selectivity indices of these four active compounds against Vero cells are provided in Table 1, where 1083 and lactoferricin exhibited relatively high SI values of 10.7 and 4.9, respectively.

In the case of MNV-1, all four peptides (melimine, Mel4, LL-37, and lactoferricin) exhibited strong activity with relatively lower IC_50_ values of 4.8 µM, 8.6 µM, 4.2 µM, and 23.18 µM, respectively (Figure 4B; Table 1). LL-37 and lactoferricin demonstrated low toxicity to Raw 246.7 macrophages and emerged as a potent and non-toxic antiviral agent against norovirus, with SI values of 11.8 and 11.1, respectively.

### 2.2. Indirect Inactivation

To explore additional mechanisms of action of the tested compounds, their indirect inactivation characteristics were evaluated using pre-treatment and post-treatment assays. No significant antiviral activity was observed in either assay for either enveloped or non-enveloped viruses.

### 2.3. Transmission Electron Microscopy Analysis

**Figure 5:**
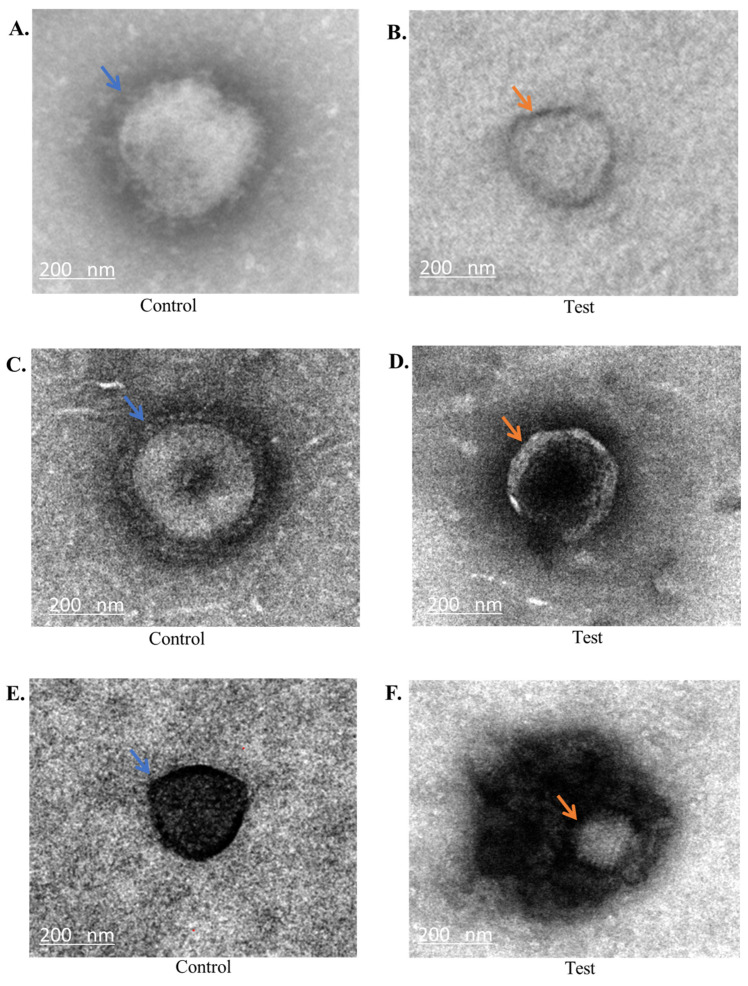

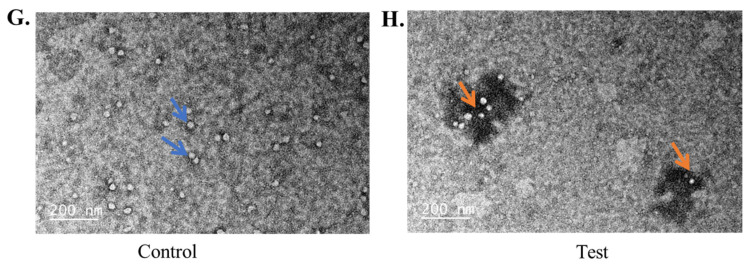
TEM images of viruses. (**A**) Untreated H1N1 virus, with blue arrows indicating the presence of the outer membrane. (**B**) H1N1 virus treated with peptide mimic 610, with orange arrows indicating the absence of the outer layer and reduction in the overall size of the viral particles. (**C**) Untreated H3N2 virus with blue arrows pointing to the outer spikes in the envelope. (**D**) H3N2 virus treated with peptide mimic 610, with orange arrows indicating a naked capsid without the outer spikes and envelop. (**E**) Untreated HAdV-5, with a blue arrow highlighting the intact capsid. (**F**) HAdV-5 virus treated with Mel4, where the orange arrow indicates a smaller, less electron-dense capsid surrounded by a large electron-dense area. (**G**) Untreated MNV-1, with blue arrows highlighting the small virus particle. (**H**) The impact of Mel4 on MNV-1, with the viral particles being surrounded by a large, electron-dense area (orange arrows).

The findings from the previous assays indicated that both the peptide and mimics directly act on the viruses. Hence, each virus was exposed to one of its most active peptides or mimics for 2 h, and the impact was assessed using transmission electron microscopy. H1N1 treated with mimic 610 resulted in a dramatic reduction in the average particle size of the virus from 120 nm to 48 nm (Figure 5A,B) with disruptions to the envelope and spike proteins. Similarly, when the H3N2 virus was treated with mimic 610, there was a reduction in size and disruption to the envelop and spike proteins, leaving behind a naked capsid structure (Figure 5C,D). Upon exposure of the non-enveloped virus HAdV-5 to the peptide Mel4, there was a decrease in capsid size from 80 nm to 57 nm, accompanied by an increased accumulation of black debris surrounding the capsid (Figure 5E,F). Similarly, treatment of MNV-1 with the Mel4 resulted in a reduction in capsid size from 26–33 nm to 7–16 nm, along with the presence of background debris surrounding the capsids (Figure 5G,H).

### 2.4. Conformational Analysis of Mel4 and 758

**Figure 6:**
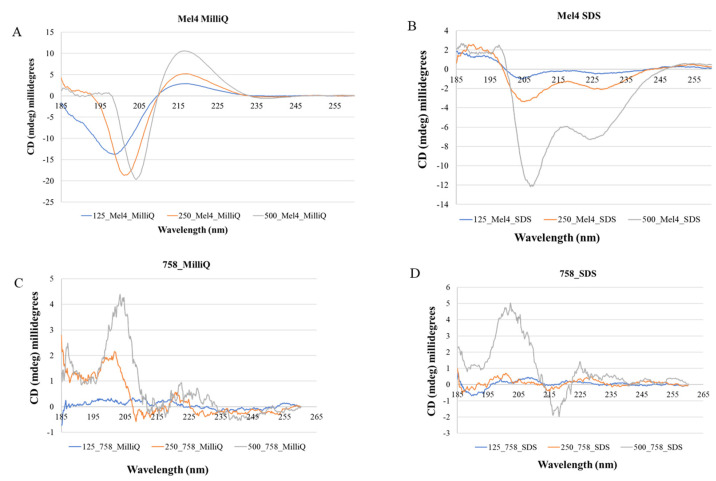
Circular dichroism spectra of Mel4 and 758. The compounds were analyzed at three different concentrations (gray line = 500 µM; orange line = 250 µM; blue line = 125 µM) in milliQ water and 30 mM SDS.

To understand how the AMP Mel4 and mimic 758 interacted with lipid membranes, circular dichroism (CD) measurements were conducted in water and SDS (30 mM), a commonly used cell membrane mimetics. The CD analysis results are presented in Figure 6. In water, Mel4 exhibited a slight positive peak, indicating a minor degree of aggregation with a notable random coil structure (Figure 6A). In SDS, Mel4 displayed a shift in its secondary conformation, exhibiting a significant α-helical structure alongside some minor beta sheet characteristics (Figure 6B). Conversely, mimic 758 had a beta sheet-like structure in both solvents (Figure 6C,D).

## 3. Discussion

This study investigated the effectiveness and mechanisms of action of thirteen compounds, including natural AMPs, synthetic AMPs, and AMP mimics, against influenza A viruses (H1N1 and H3N2), human adenovirus 5 (HAdV-5), and murine norovirus (MNV-1). Some of the compounds were effective when added directly to the viruses, but none were active when added to host cells either before or after the attachment of viruses, indicating that the compounds acted directly on the viral particles. Interestingly, the AMP mimics were more active on the enveloped influenza viruses compared to the non-enveloped adenovirus or norovirus. Previous research demonstrated that compounds with the same backbone anthranilamide structures (called compounds 11 and 14 in the previous study) as the compounds 758, 1091, 1096, 1083, and 610 used in the current study were also active against enveloped coronavirus and Herpes simplex 1 [53].

The AMPs in our study were active against non-enveloped viruses, contrasting with previous findings that LL-37 reduces infectivity of H1N1 and H3N2 viruses [54], with IC_50_ of approximately 0.5 to 5 µM [55]. LL-37 demonstrated activity against IAV when pre-incubated with host cells or applied post-infection [54]. These differences could stem from factors such as the use of MDCK cells for H1N1 culturing in our study versus egg culturing in previous studies and the use of different H3N2 strains [54,55]. The purity of LL-37, when stated in previous studies [55], was equivalent to that used in the current study, suggesting that this was not a factor. Virus quantification methods also differed; we used TCID50, while others used qPCR, ELISA, and plaque assays. Importantly, previous studies did not neutralize LL-37’s action, whereas we used 10% FBS as a neutralizing agent. Further research is needed to clarify these factors. Additionally, unlike other studies showing bovine lactoferrin’s antiviral potential [56,57], as well as certain peptides derived from lactoferrin [58], our research found no activity against these strains. Interestingly, the lactoferricin sequence we utilized, derived from the N-lobe, aligns with studies indicating that some antiviral activity of lactoferrin is associated with the C-lobe rather than the N-lobe [58,59].

Our research found no activity for LL-37 against adenovirus type 5 after 3 h of exposure, aligning with a previous report that found no effect with 111 µM LL-37. [60]. Other studies have shown bovine lactoferrin’s antiviral potential against adenovirus, with the parent protein being more effective than the N-lobe fragment lactoferricin despite lactoferricin having a higher selectivity index [61,62]. However, the previous study reported an IC50 of 0.5 ± 0.01 mg/mL, much higher than our finding of 52.52 µM, possibly due to differences in viral strains, cell types, and methodologies [62]. Notably, the previous study [62] utilized the cyclic form of lactoferricin, whereas ours focused on the linear form.

Our research demonstrated that both natural and synthetic peptides inhibited MNV-1 virus infection in Raw 246.7 cells following a 3 h incubation with varying peptide concentrations, which is similar to one previous study [63]. Interestingly, we did not find any prior literature indicating the antiviral activity of LL-37 against MNV-1. Although there are limited studies examining the impact of the parent protein lactoferrin on human norovirus [64] and murine norovirus [65], showing its ability to inhibit viral attachment and replication, to our best knowledge, there is currently no documented evidence of lactoferricin’s activity against the MNV-1 virus.

Transmission electron microscopy revealed that peptide mimics targeted the outer envelope of influenza viruses (H1N1 and H3N2). This observation aligns with a similar pattern observed in our previous study, where these mimics also targeted the virus envelope of a coronavirus and Herpes simplex virus type 1 [53]. The current investigation into adenovirus and norovirus showed that peptides appeared to target the capsid. We could find no prior reports on these effects.

Our extended objective was to understand peptide and mimic interactions with lipid envelopes and capsid proteins of non-enveloped viruses. We examined cationic amino group placement, hydrophobic group substitution, and net charge, especially in mimics. Compounds 610 and Sau-22, despite differences in naphthyl vs. biphenyl substitution and cationic group positioning, showed strong antiviral activity against IAV, suggesting that aryl group placement may be less critical if a positive charge is sufficient. Compounds NAPL, 3-BIPL, and 4-BIPL, all with two cationic lysine groups on the same side, were active, although 3-BIPL was slightly less effective. Increasing net charge beyond a threshold did not enhance efficacy, as seen with compounds 758 (+2 charge) and 1083 (+3 charge), indicating other factors may influence activity. Peptidomimetics generally had limited activity against non-enveloped viruses, except for 1083 and NAPL, which differed primarily in charge. Identifying broad-spectrum peptide mimics that are effective against both enveloped and non-enveloped viruses remains challenging and requires significant effort.

Peptides showed higher activity against non-enveloped viruses. To comprehend how the secondary structure of peptides or mimics may relate to their antiviral activity alongside factors such as charge, hydrophobicity, and size, we conducted circular dichroism analysis for one of the peptides, Mel4, and mimic 758. The secondary structure analysis of these compounds revealed distinct behaviors. Mel4 exhibited a random coil conformation in water but transitioned to an α-helix structure in SDS [66]. Whereas mimic 758 maintained a slight beta-sheet structure in both environments, Mel4’s secondary structure and its direct targeting of non-enveloped viruses resembled a previous finding with the peptide RiLK1 (RLKWVRIWRR), which shares a similar structure with Mel4 [63,67]. Nonetheless, variations in secondary structure between compounds Mel4 and 758 may contribute to their diverse actions against different virus groups. Further research is essential to understand the precise mechanisms of action of these compounds to assess their efficacy against a broader range of viral strains along with their structure–activity relationship.

Our results suggest that the compounds, which target the outer envelope of enveloped viruses, should be effective against most viral mutants. For instance, we tested two strains of influenza A virus, H1N1 and H3N2, and our peptide mimics demonstrated similar activity against both strains. However, further laboratory testing is necessary to precisely determine the antiviral efficacy of these compounds on virus mutations.

## 4. Materials and Methods

### 4.1. Cell Culture

Madin-Darby Canine Kidney cells (MDCK) (American Type Culture Collection, ATCC-CRL-2936), Vero (African green monkey kidney cells; ATCC-CCL-81), and RAW 264.7 (ATCC TIB-71) cells were cultured in Dulbecco’s Modified Eagle Medium (DMEM; Thermo Fisher Scientific Australia Pty Ltd., Melbourne, VIC, Australia), containing 10% fetal bovine serum (FBS; Bovogen Biologicals Pty Ltd., Melbourne, VIC, Australia) and 1% penicillin–streptomycin (Thermo Fisher Scientific Australia Pty. Ltd.) at 37 °C with 5% CO_2_ enrichment.

### 4.2. Virus Infection

The influenza viruses H1N1 A/PR/8/34 (ATCC-VR-1469) and H3N2 A/Hong Kong/8/68 (ATCC-VR-1679) were propagated in MDCK cells with the addition of 2 μg/mL Tosylamide Phenylethyl Chloromethyl Ketone-treated Trypsin (Thermo Fisher Scientific Aust Pty Ltd.). Human Adenovirus type 5 (HAdV-5; ATCC-VR-5) was cultured on Vero cells, and murine norovirus type 1 (MNV-1; ATCC-VR-1937) was cultured on RAW 264.7 using DMEM media supplemented with 10% FBS and 1% penicillin–streptomycin. Virus stocks were prepared and stored at −80 °C. Murine norovirus (MNV) possesses structural and genetic similarities to HuNoV and is frequently utilized as a surrogate model [68,69,70].

### 4.3. Peptides and Mimics

Melimine and Mel4 peptides were purchased from AusPep Peptide Company (Tullamarine, VIC, Australia) with a minimum purity of 90%. Additionally, two other peptides (LL-37 and lactoferricin) were acquired from Synpeptide (Shanghai, China) with a purity of >95%. Peptide mimics (758, 1091, 1096, 1083, 610, NAPL, 3-BIPL, 4-BIPL, and Sau-22) were synthesized using established protocols and published in previous papers and patents (WO2018081869A1 and Australian Provisional Patent Application No. 2021902457) [52,53,71]. The structures of the compounds are provided in Table 2. All compounds were dissolved in dimethyl sulfoxide (DMSO) to prepare stock solutions. Subsequently, these stocks were diluted in DMEM to achieve the desired concentrations for experimentation. The volume of DMSO associated with the tested concentrations of peptides or mimics was evaluated individually, and it was determined that DMSO was not active against the viruses.

### 4.4. Antiviral Testing

The antiviral activity of the peptides and mimics was evaluated using three distinct assays: virus pre-treatment, cell pre-treatment, and post-infection cell treatment, each varying in the timing of compound application. The overall antiviral methodology was adapted from published sources [53,72,73,74] with several modifications. Preliminary experiments identified 2 h or 3 h exposure times as optimal for the antiviral effects of the compounds in these assays. For H1N1, H3N2, and MNV-1, the TCID_50_/mL assay was employed, whereas for adenovirus, the plaque assay (using 1% crystal violet as the disclosing stain) [53] was employed. Cells were seeded in 24-well or 96-well plates at a density of 1 × 10^6^ cells/mL, reaching 80–90% confluency within 24–48 h for subsequent experiments. All assays utilized compounds in DMEM medium without FBS. Each experiment was performed in duplicate, and three independent experiments were performed. The inhibitory effect on viral infectivity was determined by comparing TCID_50_/mL and PFU/mL values for the test and control.

Direct Inactivation: Viruses were incubated with different concentrations of compounds for 3 h. After incubation, the mixtures were diluted in DMEM with 10% FBS to neutralize the peptides or mimics. The dilutions were added to cell monolayers for 1 h, followed by the addition of an agar overlay containing 1% agar for plaque assay or fresh media for TCID_50_/mL and kept at 37 °C for 72 h [53].

Cell Pre-treatment: Host cells were treated with the test compounds for 3 h at 37 °C. Subsequently, the cells were rinsed twice with 1X phosphate buffer saline (PBS) solution (composed of 8 g NaCl, 0.2 g KCl, 1.44 g Na_2_HPO_4_, and 0.24 g KH_2_PO_4_ per liter of water). Following the rinsing step, the virus was introduced to allow infection to take place for 1 h at 37 °C. Any excess or unbound virus was then removed by washing twice in PBS, and either an agar overlay containing 1% agar for the plaque assay or fresh media for TCID_50_/mL was added and incubated for another 72 h at 37 °C [53].

Post-viral infection: The cells were exposed to viruses for 2 h at 37 °C to allow them to enter cells. Subsequently, any excess viral cells were removed by washing twice in PBS, and test compounds were introduced for another 3 h at 37 °C. Afterward, the solutions containing the compounds were discarded, and the cells were washed twice with 1X PBS. Following this, either an agar overlay containing 1% agar for the plaque assay or fresh media for TCID_50_/mL was added and kept at 37 °C for 72 h [53].

The assay demonstrating the highest antiviral activity for most viral models was chosen, and GraphPad Prism software was used for a non-linear regression analysis to determine the IC_50_ (concentration required for 50% inhibition) value.

### 4.5. Cytotoxicity Assay

The 3-(4,5-dimethylthiazol-2-yl)-2,5-diphenyltetrazolium bromide assay (MTT assay) [53,69] was used to assess the cytotoxicity of compounds against the viral host cells (MDCK, Raw 246.7, or Vero). This assay relies on the reduction of the yellow MTT compound to a dark blue formazan product by viable and metabolically active cells. Viral host cells were cultured in 96-well plates and incubated at 37 °C in a 5% CO_2_ atmosphere and then treated with various concentrations of the test compounds together with further incubation for 24 h. After incubation, 100 μL of a 5 mg/mL MTT solution was added to each well and incubated for 2–4 h at 37 °C. The supernatant was then discarded, and 100 μL of 100% DMSO was added to dissolve the formazan salts with vigorous agitation for 10 min at room temperature. The absorbance at 570 nm was measured along with 630 nm as a reference or background control using a spectrophotometer. Cytotoxicity was determined by comparing the absorbance values in the test and control wells expressed as a percentage. A total of 100 μL DMSO was used for the negative control, while 100 μL of culture medium represented the positive control. All experiments were repeated three times, and the mean values with standard error were reported. Non-linear regression analysis was performed using GraphPad Prism software to determine the CC_50_ (concentration at which 50% of cells are killed).

### 4.6. Transmission Electron Microscopy (TEM)

The viruses were exposed to the peptide/mimics for 2 h at 37 °C. Following this, 10 µL of the treated mixture was applied onto a glow-discharged carbon-coated 200 mesh copper grid and left to evaporate for 5 min. Subsequently, the sample was stained with 2% phosphotungstic acid (pH 4.5) for 1 min to enhance contrast. After air-drying, the grids were examined using an FEI Tecnai G2 20 transmission electron microscope (TEM) [53].

### 4.7. Circular Dichroism

Circular dichroism (CD) analysis was conducted using the Jasco J-1500 spectropolarimeter to assess the secondary structure of peptides and mimics. The samples were placed in a quartz cuvette with a path length of 0.1 cm (Fisher brand, Thermo Fisher Scientific Aust Pty Ltd.). Spectra were recorded in the range of 185–260 nm at a scan speed of 200 nm/min, averaging 4 scans. The impact of the solvents milliQ water or 30 mM SDS on Mel4 and mimic 758 was evaluated at three concentrations: 500 µM, 250 µM, and 125 µM. Data were averaged, and the spectrum of a sample-free control was subtracted. Alpha (α) helix structure was identified by 2 negative bands, one at around 208 and another at around 222 nm and a positive band at 190 nm; beta sheet structure by a negative band at 218 nm and a positive band at 196 nm; and random coil structure by a positive band at 212 nm and a negative band at approximately 195 nm [75,76].

### 4.8. Statistical Analysis

The data were presented as the average ± standard error of the mean (SEM) from a minimum of three separate experiments. Statistical analysis was conducted using GraphPad Prism 9.5.0 software with a one-way ANOVA followed by Dunnett’s multiple comparisons test, using a 95% confidence interval. Statistical significance was defined as a *p*-value below 0.05 (* *p* < 0.05. ** *p* < 0.01. *** *p* < 0.001. ns, not significant) [77,78].

## 5. Conclusions

Natural and synthetic peptides showed no effectiveness against H1N1 and H3N2 strains, while peptide mimics exhibited notable antiviral activity against both strains. AMPs were effective against norovirus, and both AMPs and mimics displayed some activity against adenovirus. It is likely that both AMPs and mimics acted directly on virus particles, as they were not effective when cells were treated beforehand, or compounds were added post-viral entry. Various antiviral assessments and electron microscopy images obtained through TEM collectively demonstrated that AMP mimics could target the envelope of influenza viruses, while AMPs targeted the capsid of adenovirus and norovirus. These AMPs and mimics hold promise for further exploration in the development of effective antiviral strategies against a range of viral infections.

## Figures and Tables

**Table 1:** The IC_50_, CC_50_, and selectivity indices for all compounds against influenza A (H1N1 and H3N2), human adenovirus type 5, and murine norovirus 1 (bold font indicates most active compounds with greatest selectivity indices).

	Virus/Host Cell	Influenza A Virus (H1N1)/MDCK			Influenza A Virus (H3N2)/MDCK			Human Adenovirus-5/Vero			Murine Norovirus-1/Raw 246.7		
	Peptide/Mimics	IC_50_ (µM)	CC_50_ (µM)	SI	IC_50_ (µM)	CC_50_ (µM)	SI	IC_50_ (µM)	CC_50_ (µM)	SI	IC_50_ (µM)	CC_50_ (µM)	SI
1	**Melimine**	>500	28.07	-	>500	28.07	-	>125	14.5	-	4.8	0.03	0
2	**Mel4**	>500	12.67	-	>500	12.67	-	47.43	47.6	1	8.6	30.27	3.5
3	**LL-37**	>500	2.8	-	>500	2.8	-	>125	36.74	-	**4.2**	**49.9**	**11.8**
4	**Lactoferricin**	>500	3.9	-	>500	3.9	-	52.52	257.9	4.9	23.18	258.7	11.1
5	**758**	12.34	147.1	11.9	**9.3**	**147.1**	**15.8**	>62.5	151.8	-	>62.5	16.2	-
6	**1091**	23.09	127.9	5.5	24.26	127.9	5.2	>62.5	105.4	-	>62.5	45.60	-
7	**1096**	22.22	47.21	2.1	24.01	47.21	1.9	>62.5	57.29	-	>62.5	4.7	-
8	**1083**	28.50	250.5	8.7	27.65	250.5	9.05	**45.15**	**484.7**	**10.7**	>62.5	75.54	-
9	**610**	**2.35**	**33.30**	**14.1**	3.7	33.30	9	>62.5	16.7	-	>62.5	13.9	-
10	**NAPL**	10.49	111.8	10.6	10.51	111.8	10.6	59.27	96.1	1.6	>62.5	40.04	-
11	**3-BIPL**	19.24	28.11	1.4	18.63	28.11	1.5	>62.5	27.8	-	>62.5	28.24	-
12	**4-BIPL**	10.34	12.78	1.2	10.4	12.78	1.2	>62.5	38.8	-	>62.5	60.18	-
13	**Sau-22**	6.93	79.43	11.4	5.34	79.43	14.8	>62.5	15.18	-	>62.5	9.3	-

**Table 2:** Detail structure, molecular mass, and charges of all tested peptides and mimics.

LL-37—molecular mass 4493.3; charge +6 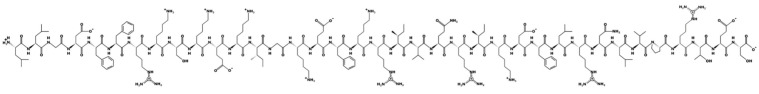		
Melimine—molecular mass 3786.6; charge +15 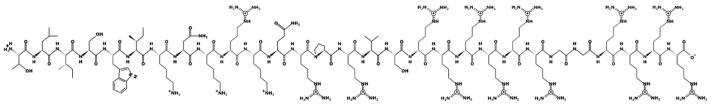		
Mel4—molecular mass 2347.8; charge +14 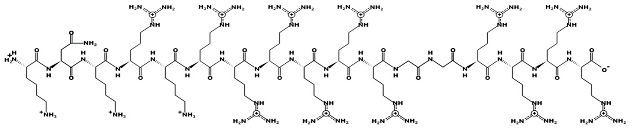		Lactoferricin—molecular mass 1544.9; charge +4 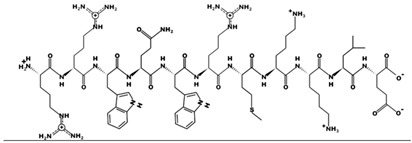
758—molecular mass 759.71; charge +2 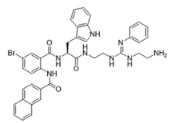	1091—molecular mass 601.51; charge +1 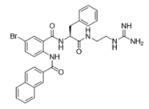	1096—molecular mass 720.67; charge +2 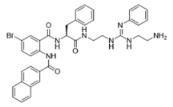
1083—molecular mass 726.68; charge +3 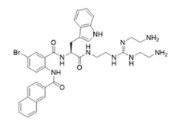	610—molecular mass 491.60; charge +2 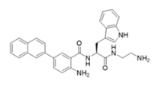	NAPL—molecular mass 726.68; charge +2 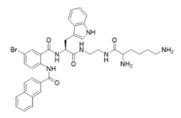
3-BIPL—molecular mass 752.71; charge +2 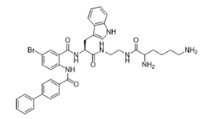	4-BIPL—molecular mass 752.71; charge +2 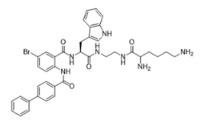	Sau-22—molecular mass 745.68; charge +2 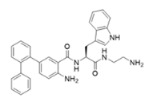

## Data Availability

Data are contained within the article and available upon request.
